# Ultrastructural Features of Neurovascular Units in a Rat Model of Chronic Compressive Spinal Cord Injury

**DOI:** 10.3389/fnana.2017.00136

**Published:** 2018-01-10

**Authors:** Jinghui Xu, Houqing Long, Wenli Chen, Xing Cheng, Haoyang Yu, Yangliang Huang, Xiaobo Wang, Fobao Li

**Affiliations:** ^1^Department of Spine Surgery, The First Affiliated Hospital of Sun Yat-Sen University, Guangzhou, China; ^2^Guangdong Provincial Key Laboratory of Orthopedics and Traumatology, The First Affiliated Hospital of Sun Yat-Sen University, Guangzhou, China

**Keywords:** neurovascular unit, ultrastructure, chronic spinal cord compression, rat, animal model

## Abstract

Chronic spinal cord compression is the most common cause of spinal cord impairment worldwide. Objective of this study is to assess the ultrastructural features of the neurovascular unit (NVU) in a rat model of chronic compressive spinal cord injury, 24 *SD* rats were divided into two groups: the control group (*n* = 12), and the compression group (*n* = 12). A C6 semi-laminectomy was performed in the control group, whereas a water-absorbent polyurethane polymer was implanted into the C6 epidural space in the compression group. The Basso Beattie Bresnahan (BBB) scores and the somatosensory evoked potentials (SEP) were used to evaluate neurological functions. Transmission Electron Microscopy (TEM) was performed to investigate the change of NVU at the 28th day after modeling. Compared with the control group, the compression group shows a significant reduction (*P* < 0.05) of BBB score and a significant severity (*P* < 0.05) of abnormal SEP. TEM results of the compression group showed a striking increase in endothelial caveolae and vacuoles; a number of small spaces in tight junctions; a significant increase in pericyte processing area and vessel coverage; an expansion of the basement membrane region; swollen astrocyte endfeet and mitochondria; and the degeneration of neurons and axons. Our study revealed that damage to NVU components occurred followed by chronic compressive spinal cord injury. Several compensatory changes characterized by thicker endothelium, expansive BM, increased pericyte processing area and vessel coverage were also observed.

## Introduction

Chronic spinal cord compression is the most common cause of spinal cord impairment worldwide. Cervical spondylosis myelopathy is characterized by progressive stenosis of the cervical canal and compression of the spinal cord due to the herniated cervical discs and degenerative changes. Diseases such as ossification of the posterior longitudinal ligament (OPLL), spinal tuberculosis, extramedullary tumor and other degenerative deformities also cause spinal cord compression (Fehlings et al., 2013; Kalsi-Ryan et al., 2013; Karadimas et al., 2013a). Previous studies suggest that mechanical compression of the spinal cord could cause ischemia, inflammation (Song et al., 2015), neuronal apoptosis and disruption of the blood-spinal cord barrier (Karadimas et al., 2015). However, most ultrastructural evidence of NVU is from acute spinal cord injury (Kaptanoglu et al., 2002; Smith and Jeffery, 2006; Ramadan et al., 2017) or brain ischemia (Nahirney et al., 2016). In chronic spinal cord compression, the ultrastructural changes of neurovascular units (NVU), which include BSCB, neurons and some related cells have not been investigated thoroughly until now (Frascarelli et al., 1990).

The neurovascular unit (NVU) is a specialized structure comprised of vascular endothelium, pericytes, astrocytes, neurons, and, in a broader context, the extracellular matrix (Muoio et al., 2014). This structure plays a crucial role in maintaining normal homeostasis in the spinal parenchyma, and serves as an important defense during chronic compression and other pathologies (Muoio et al., 2014). Abnormal alteration within the NVU has implications for co-existing diseases such as hypertension or diabetes that have a negative effect on spinal microcirculation. The concept of the NVU highlights the importance of interactions between blood vessels and other spinal parenchymal cells. Therefore, assessing the ultrastructure features of NVU in the chronic compressive segment of the cervical cord could help to clarify interactions among various components of the unit and to determine which structural change is responsible for the high permeability and functional neural deficits, and could possibly shed light on new and inclusive therapies for chronic spinal cord injury diseases.

Currently, investigations regarding the chronic compression of the cervical spinal cord focus more commonly on ischaemic-hypoxic injury, inflammation, neuronal apoptosis and disruption of the blood-spinal cord barrier (BSCB). Evidence supporting the disruption of NVU mainly come from functional tests, light microscopic or biochemical experiments showing changes in neurological function, EB penetrability (Evans blue assay for visualizing BSCB permeability), protein immunofluorescence or protein/mRNA levels after chronic injury (Karadimas et al., 2015). Meanwhile, to our knowledge, little evidence of NVU disruption in this condition as evidenced by TEM has been reported (Karadimas et al., 2013b). Over the past few decades, most of the TEM attention has focused on blood brain barrier (BBB) (Frontczak-Baniewicz et al., 2011) and acute spinal cord injury (Kaptanoglu et al., 2002).

Our previous study using a rat model indicated that the most serious neurological damage occurred at the 28th day after chronic compression (Cheng et al., 2015) and was accompanied by reduction of micro vessels in the dorsal gray matter of cervical cord (Long et al., 2014; Cheng et al., 2015). Simultaneously, high permeability along with flattening and widening of the compressive zone/segment was found (Long et al., 2014). All evidence showed that the worst lesion of neurons was observed at the 28th day after compression.

Collectively, one important and unresolved knowledge gap in the existing literature is how the ultrastructure is altered after chronic spinal cord compression. Chronic compression is known to produce subtle morphological and functional changes to the NVU, and TEM has been used as a powerful tool to explore ultrastructure in many specimens (Herrera, 1992; Hernandez-Chavarria, 2002; Kubota, 2015). Therefore, in the present study, electron microscopy (TEM) was used to assess the ultrastructure of the neurovascular unit (NVU) in the compression cervical cord segment on the 28th day after compression. Taking advantage of the clarity of electron micrographs, we may directly observe the interactions among various components of the NVU in a rat model of chronic cervical spinal cord compression. Thus, these data may provide further NVU morphological evidence and help clarify the pathophysiology of spinal cord chronic compression.

## Materials and methods

### Animal model

The experiment procedure was approved by the Research Ethics Committee of the authors' institute. A total of 24 adult male Sprague-Dawley (SD) rats (300–350 g, Experimental Animals Centre of Southern Medical University) were allocated to two groups: the control group (*n* = 12) and the compression group (*n* = 12).

In the compression group, all rats were anesthetized with 2% isoflurane with oxygen and N_2_O. The C5 lamina was exposed, then the ligamentum flavum and partial lamina were removed to access the epidural space. A water-absorbent and expandable polyurethane polymer compression sheet was implanted into the C6 epidural space on the posterolateral side to induce compression to the spinal cord. The sustained-release membrane was made of polyurethane synthesized in the laboratory from isocyanates and polyols (Guangzhou Fischer Chemical Co., Ltd., Guangzhou, China). In previous studies this compression material showed no inflammatory reaction or tissue granulation after implantation (Long et al., 2013). After complete haemostasis, the incision was closed in layers in the usual manner. For rats in the control group, the C5 lamina was exposed, then the ligamentum flavum and partial lamina were removed to access the epidural space, without inserting the compression sheet into the C6 epidural space. All animals were given an intramuscular injection of Penicillin G (8,000 U/100 g, intramuscular injection) to prevent infection post-surgery. After surgery, animals were individually housed in cages and allowed free access to food and water. Post-operative analgesia was administered as subcutaneous injection of buprenorphine (0.01 mg/kg) every 12 h for 3 days. In this study, rats needed no analgesia after 3 days.

### Motor function and neurophysiological monitoring

The Basso Beattie Bresnahan (BBB) score was used to assess the severity of paralysis due to spinal cord compression in terms of motor function. For the three groups, BBB scores were evaluated each day from 3 days pre-surgery to the 28th day post-surgery. The evaluation process was double-blinded, and the average scores in each group were calculated. In addition, all rats received the Somatosensory Evoked Potential (SEP) test before euthanasia on the 28th day (Long et al., 2013).

### Euthanasia and tissue preparation

All the rats were anesthetized with chloral hydrate (400 mg kg^−1^) prior to phrenic nerve surgery. A 3–4 cm midline incision was made on the anterior aspect of the neck from the larynx to the manubrium of the sternum. The left sternoclavicular joint was incised and the clavicle was retracted laterally to expose the phrenic nerve lying anterior to the brachial plexus. The nerve was freed from its membranous sheath and transected with microscissors. Dry horseradish peroxidase (HRP) crystals (0.5 mg, Sigma, type VI) were applied directly to the end of the transected central stump of the nerve. Care was taken to avoid injury to any other nerve in the surgical field. After 10–15 min, the wound was closed with silk sutures and the animals were allowed to survive for 48 h.

Euthanasia of all rats was achieved with deep isoflurane anesthesia and perfusion was not performed to avoid mechanical disruption of blood capillaries. The cervical spinal cords were removed and fixed by immersion in 4% paraformaldehyde (PFA) in 0.1 M phosphate buffer (PB), pH 7.2, for 16–24 h at 4°C.

Next, the cords in cervical (C5–C6) spine Were separated, and 1 mm slices fixed overnight in 2% glutaraldehyde in 0.1 M PB (Electron Microscopy Sciences, Inc., Hatfield, PA) at 4°C. On the following day, the above buffer was changed, and the tissues were post-fixed in 1% osmium tetroxide for 1.5 h, dehydrated in ethanol solutions and embedded in Epon overnight, ready for further processing.

### Electron microscopy

Ultra-thin sections of spinal cord tissue from C5-6 (The sample lays within the left gray matter in the dorsal horn under C6) measuring 70 nm to 90 nm thick were cut with an ultramicrotome (Reichert E, Co., Vienna, Austria) and stained with uranyl acetate for 10 min, followed by staining with lead citrate for 6 min. Sections were examined under a TEM (Philips CM 10, Eindhoven, Holland). Images were obtained with a Gatan SC-1000 digital camera. Calibration of images was performed by imaging a carbon replica grid (0.463 mm intervals) at the same magnifications. All chemicals used for electron microscopy processing were obtained from Electron Microscopy Sciences.

**Figure 1:**
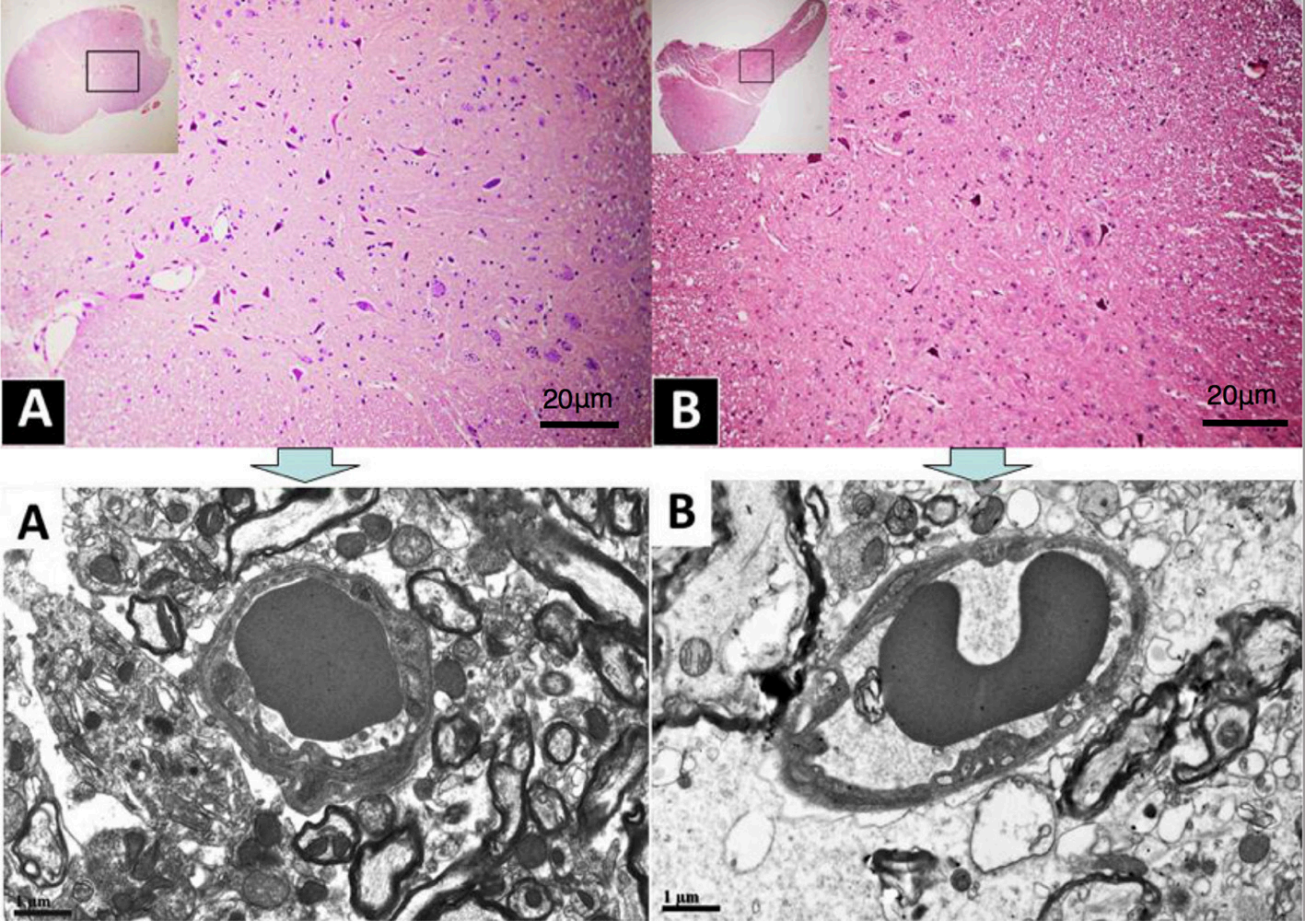
**(A,B)**, Histological sections photographed at the light microscope. Rectangles in the insets illustrate position and size of the sample examined at the TEM (pictures above), located in the dorsal horn under C6.

Ultra-thin sections were obtained from Epon blocks of the two groups. Three sections per block, per animal, was photographed at the EM, covering an area of 5.544 × 3.603 square micro-meters. Each image was examined for morphometric evaluation (Figure 1).

HRP-labeled phrenic motor neurons and their processes were identified under a light microscope, trimmed from their surrounding tissue, osmicated, dehydrated, and embedded in Araldite. One micrometer thick sections were cut from the plastic blocks and stained with toluidine blue for light microscopic examination. Ultrathin sections were obtained from those blocks which yielded heavily labeled neurons in the 1 μm thick sections. In order to confirm the presence of HRP reaction product in phrenic motor neurons, unstained ultrathin sections from each animal were first examined with the electron microscope. Adjacent sections were stained with lead citrate and uranyl acetate only when the presence of HRP was confirmed on the unstained sections.

### Data analysis

For quantitative analysis, electron micrographs (3,696 × 2,420 pixels, 5.544 × 3.603 μm,1.5 nm/pixel,) of capillaries from three groups were analyzed using Image pro plus software.

Inclusion criteria for microvessel analysis were as follows: the diameter was <8 mm, the lumen was not occluded by blood cells or plasma, and the vessel was in gray matter and within 300 mm of the compression site. Images with an endothelial or pericyte nucleus were excluded from the analysis because they generate extreme measurements (e.g., for endothelial or pericyte area/coverage) and are too infrequent to represent samples adequately. Endothelium, pericyte, and astrocytes around the microvessel were manually traced by an experimenter blind to conditions and threshold to calculate area and perimeter. Vacuolation of the endothelium was quantified by normalizing the number of vacuoles to the circumference of the vascular endothelium. Pericyte coverage of endothelial cells (reported as percentage) was calculated based on the total length of the inner pericyte processes around each vessel relative to the perimeter of the endothelium. The diameter of the capillary lumen, vacuoles, or caveolae, as well as basement membrane thickness, was measured in straight line segments at four cardinal points in the microvessel or organelle. Any endothelial tight junctions (TJ) with a fluid space >50 nm wide was classified as an intercellular space.

Statistical comparisons were conducted with a two-way ANOVA followed by *post-hoc* Bonferroni corrected *t*-tests to compare dependent measurements. All statistical tests were calculated based on means generated from the number of animals in each group. *P* < 0.05 were considered to be statistically significant. The results are expressed as the mean ± standard error of the mean.

## Results

### BBB score

**Figure 2:**
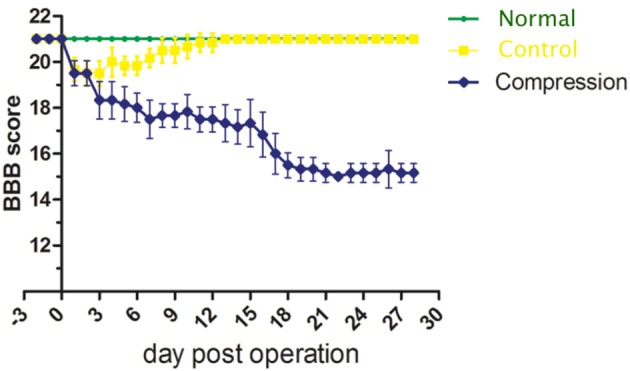
Plot of BBB score averages from the normal, control and compression groups.

Based on the analysis of BBB scores, neurological function in the compression group showed a significant decline after surgery (Figure 2). On the 28th day, BBB scores averaged 21.0 ± 0.0 in the control group, and 15.5 ± 0.224 in the compression group. There was significant difference between two groups (*P* < 0.05).

### SEP

**Figure 3:**
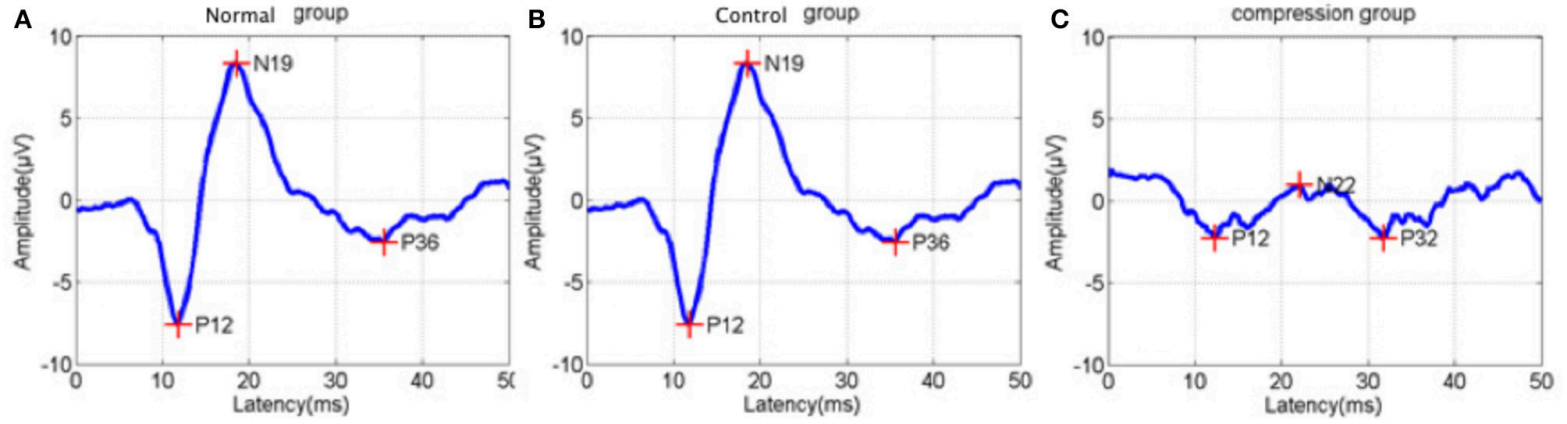
Sample of latency prolongation and amplitude reduction of somatosensory evoked potentials (SEP) from normal status **(A)**, control status **(B)**, and compression status **(C)**.

In addition to BBB assessment, SEP tests were performed in the two groups to evaluate functional changes after surgery. Amplitude in the compression group were significantly reduced, while latencies increased compared to the control group (Figure 3, Table 1, *P* < 0.05).

**Table 1:** Average amplitude and latency of the three groups (^*^P < 0.05).

**Group**	**Latency (ms)**	**Amplitude (μV)**
Normal (*n* = 3)	4.26 ± 0.22	7.17 ± 0.11
Control (*n* = 12)	4.29 ± 0.25	7.22 ± 0.23
Compression (*n* = 12)	8.47 ± 0.35^*^	3.22 ± 0.23^*^

### Transmission electron microscope examination

#### Endothelium and TJ

**Figure 4:**
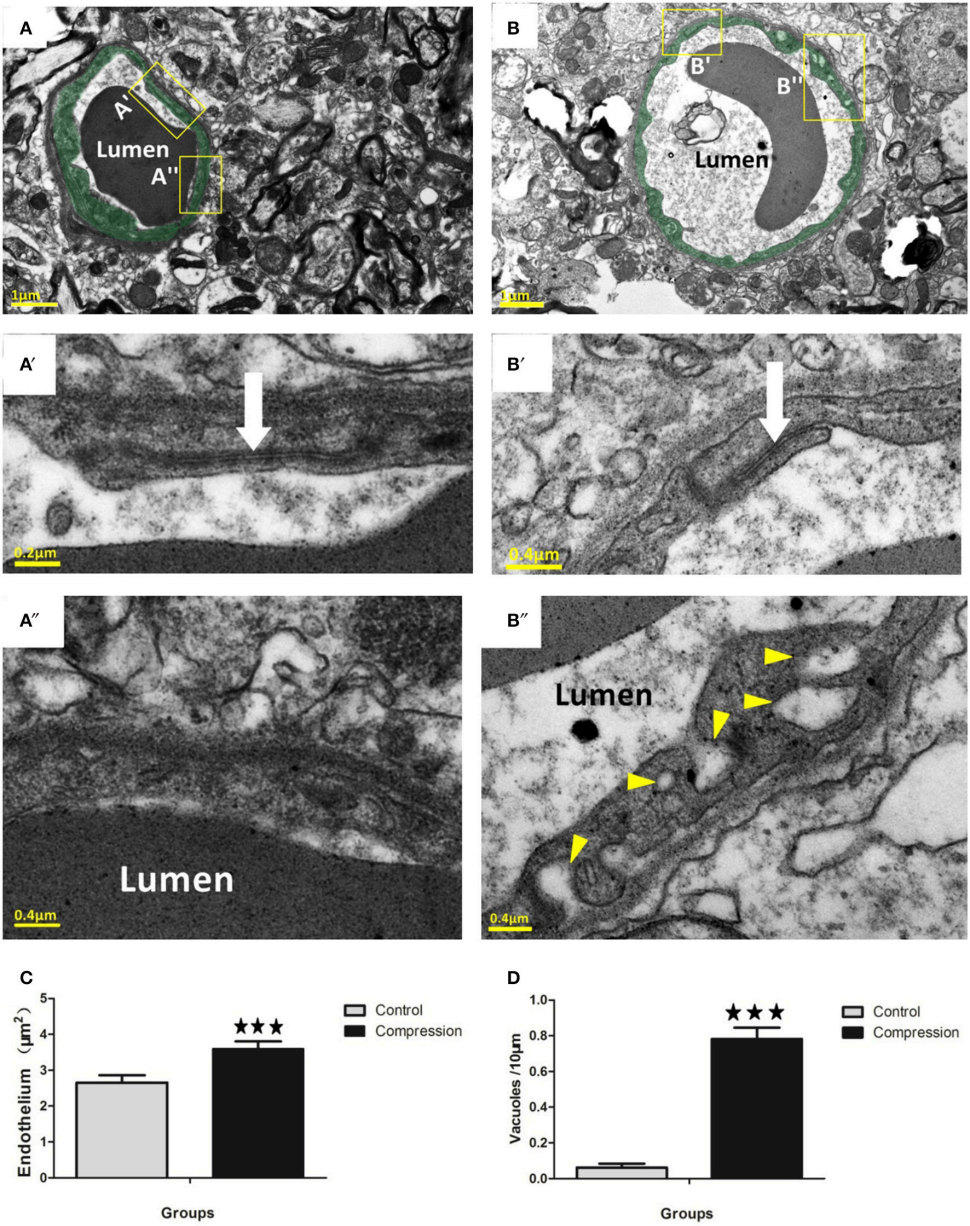
Electron micrographs (EMs) of capillaries in the control group **(A)**. Endothelium is lightly shaded as green **(A,B)**. Insets **(A**′**, A**″**)** below the image shows a relatively thin endothelial layer **(A**″**)** and intact TJs (white arrow in **A**′). EMs of capillaries in the compression group **(B)**. Insets **(B**′**,B**″) show that the slightly swollen endothelium was densely packed with putative caveolae-like vesicles (arrowheads) on the luminal and abluminal sides. Several TJs in the compression group (white arrows in **B**′) were still intact. Histograms show that endothelial thickness **(C)** and vacuole formation **(D)** were significantly increased in the compression group. Vacuoles are expressed as vacuoles per 10 mm of the vascular endothelium circumference. The number of capillaries analyzed per group was 30, sampled from 12 rats per group. ^***^P < 0.001 relative to controls.

**Figure 5:**
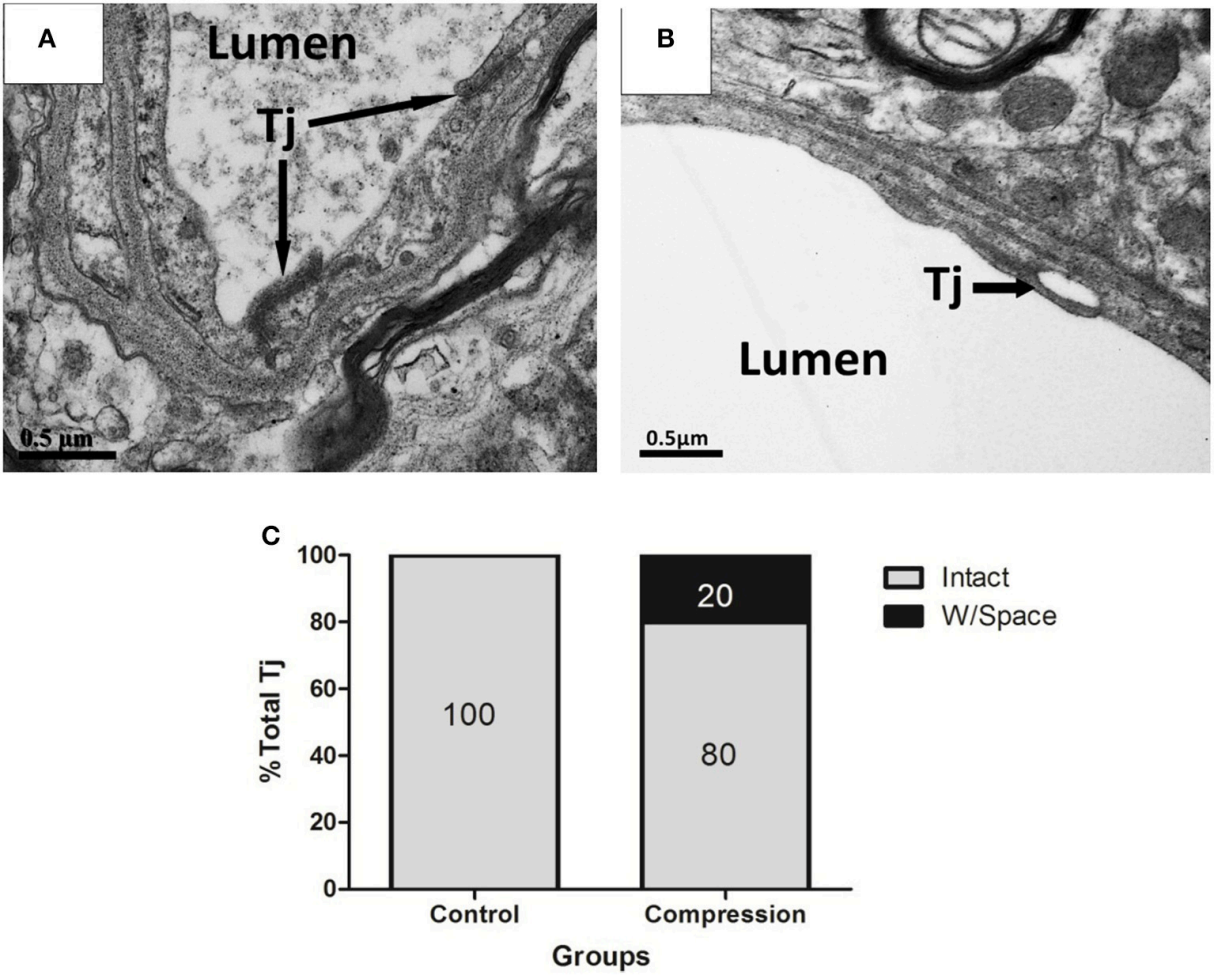
EMs of TJs in ECs from control **(A)**, and compression group **(B)**. Structurally abnormal TJs that contain large gaps (black arrows) within compressed regions **(B)**. Histogram shows the percentage of intact TJs in the two groups **(C)**. Percentages were based on the analysis of 30 TJs from 12 rats per group. Tj, tight junction.

In non-compression control micro-vessels, the endothelium was relatively thin (140 ± 50 nm thick in nonnuclear regions). Endothelial cells contained scattered small vesicles as well as electron dense TJs where the two endothelial cells interdigitated (Figures 4A,A′). Twenty-eight days after compression, the endothelium was packed with small caveolae-like vesicles (diameter: 55 ± 17.5 nm) that lined with both the luminal and abluminal sides of the endothelium (arrowheads in Figure 4B″). The endothelium was filled with caveolae-like vesicles and appeared quite swollen, as revealed by the significant increase in cross-sectional area of the endothelium (Figure 4C). In both groups, larger fluid-filled organelles (>100 nm diameter) which we refer to as vacuoles formed in the endothelium (e.g., see Figure 4B″). After compression, the number of vacuoles per 10 mm of vascular endothelium increased significantly (Figure 4D). The location of a vacuole could occur at any site within the endothelial TJs (Figure 4B′), and the shape appeared intact in the two groups (Figures 4A′,B′, 5A). However, in a few instances, we observed a small fluid-filled space within the junction (Figure 5B), which tended to increase after compression (Figure 5C).

#### Pericytes

**Figure 6:**
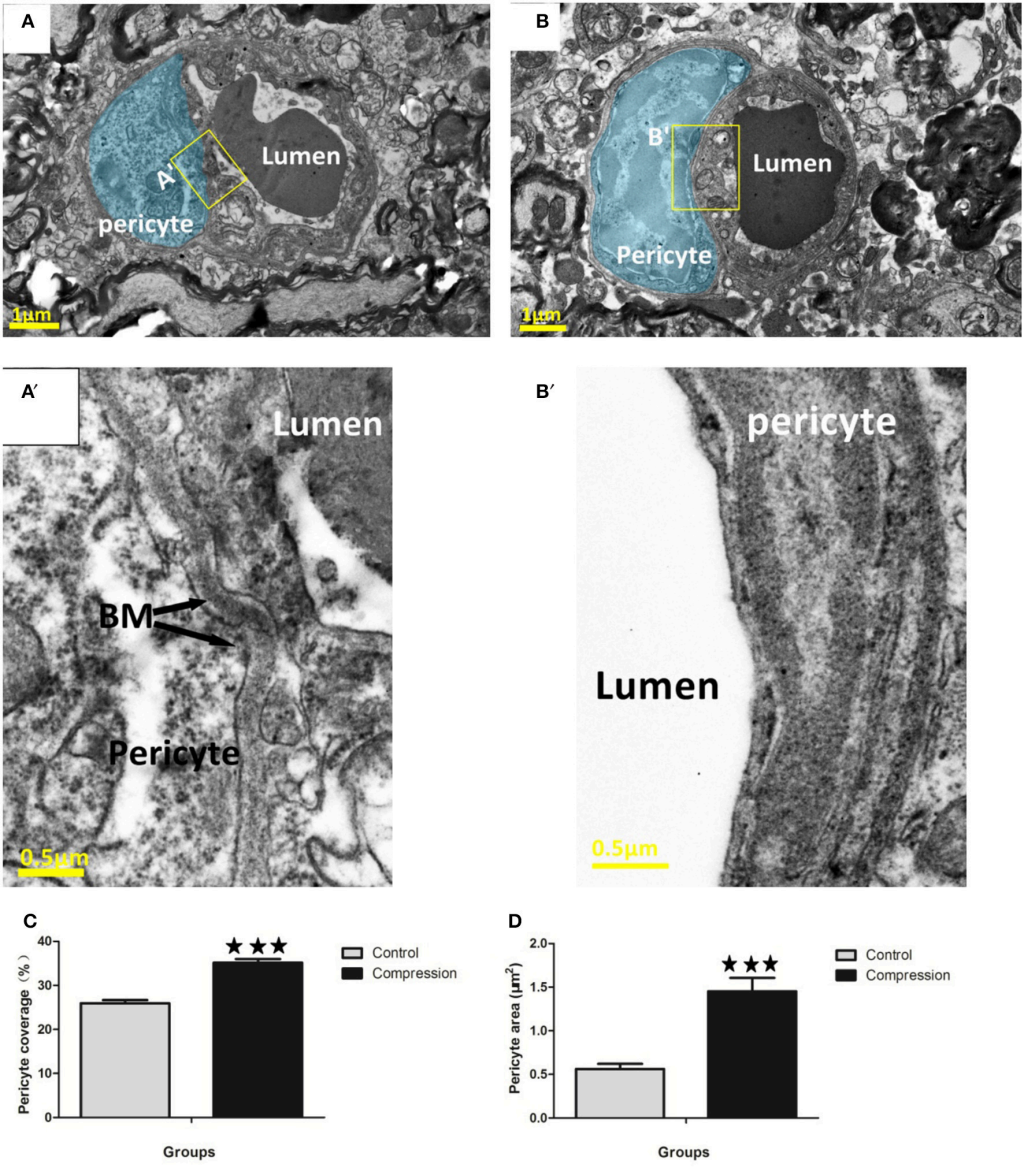
EMs of microvessels in the control group **(A,A**′**)**, and corrugated BM **(A**′**)**. Pericytes are highlighted in blue **(A,B)**. Pericyte processes appeared slightly swollen **(B,B**′**)**, and smooth BM were detected in the compression group. Quantitative analysis indicated that pericyte coverage of the endothelium **(C)** and cross-sectional area **(D)** increased significantly in the compression group. The number of capillaries analyzed per group was 30, sampled from 12 rats per group. ^***^P < 0.001 relative to controls.

Endothelial cells were enwrapped by a discontinuous layer of pericytes which are mostly ensheathed on basement membranes (Figure 6A). Pericyte processes could be distinguished by the granulated appearance of the cytoplasm (Figure 6A′). Pericyte swelling increased along with its coverage of the endothelium in the compression group. The pericytes covered 22.5–30% of the outer perimeter of the endothelium in the control group, and the percentage increased to 30.5–40.1% (Figure 6C). Pericyte area was also significantly increased in the compression group (Figure 6D). Additionally, in some examples, the vessel wall adjacent to the pericyte soma appeared constricted (Figure 6A) and was accompanied by a corrugated basement membrane (Figure 6A′), which is opposite with pericytes in the compression group that possessed a smooth vessel lumen (Figure 6B) without a corrugated appearance (Figure 6B′).

#### Astrocytes

**Figure 7:**
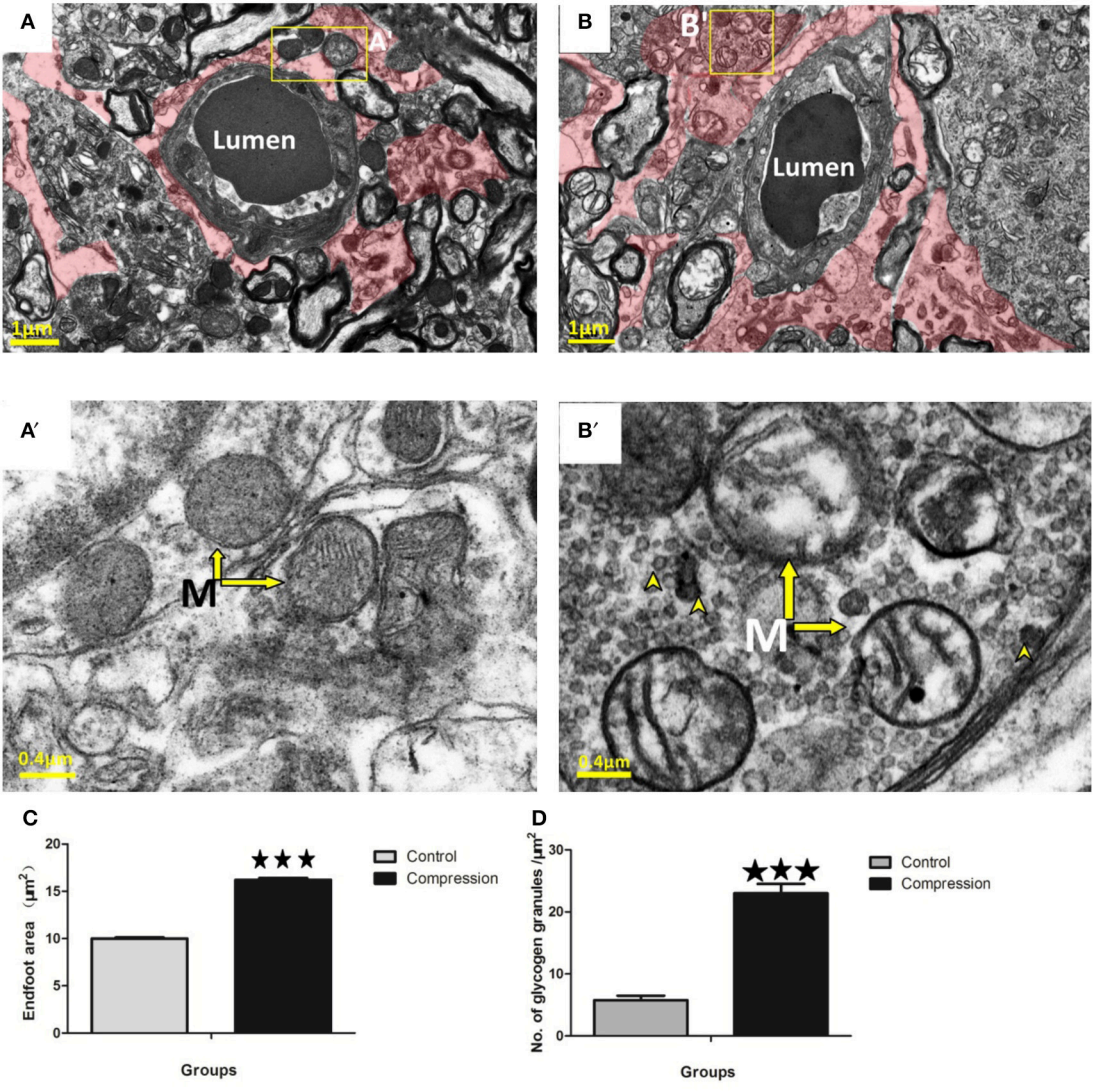
EMs of perivascular astrocytes (shaded in red) in the control group **(A)** and compression group **(B)**. Mitochondria (arrow) with normal appearance in control astrocytes **(A**′**)** or swollen mitochondria with disorganized or absent cristae in the compression group **(B**′**)**. Notably, astrocytes in the compression group were conspicuously packed with glycogen granules (see arrowheads in **B**′**,D**). Astrocyte endfoot area increased considerably after compression **(C)**. The number of glycogen granules per μm^2^ in the astrocytes increased significantly **(D)**. The number of capillaries analyzed per group was 30, sampled from 12 rats per group. M, Mitochondria; ^***^P < 0.001 relative to controls.

Astrocytes could be distinguished by the relative sparseness of electron-dense material in the cytoplasm (Figures 7A,B). Their mitochondrial membranes appear less electron-dense than those found in neurons, endothelium, and pericytes (Figure 7A′). Following compression, perivascular astrocytes underwent large-scale changes in area and remained swollen at the 28th day post-surgery (red shaded area in Figure 7B). Quantitatively, the cross-sectional area of astrocyte endfeet increased significantly after compression (Figure 7C). Mitochondria in astrocytes were also disrupted after compression, appearing swollen with partially intact cristae (arrows in Figure 7B′). Electron-dense glycogen granules (15–25 nm diameter) and rosettes (40–60 nm diameter) became abundant in the cytoplasm of astrocytes after compression (arrowheads in Figure 7B′).

#### Axons

**Figure 8:**
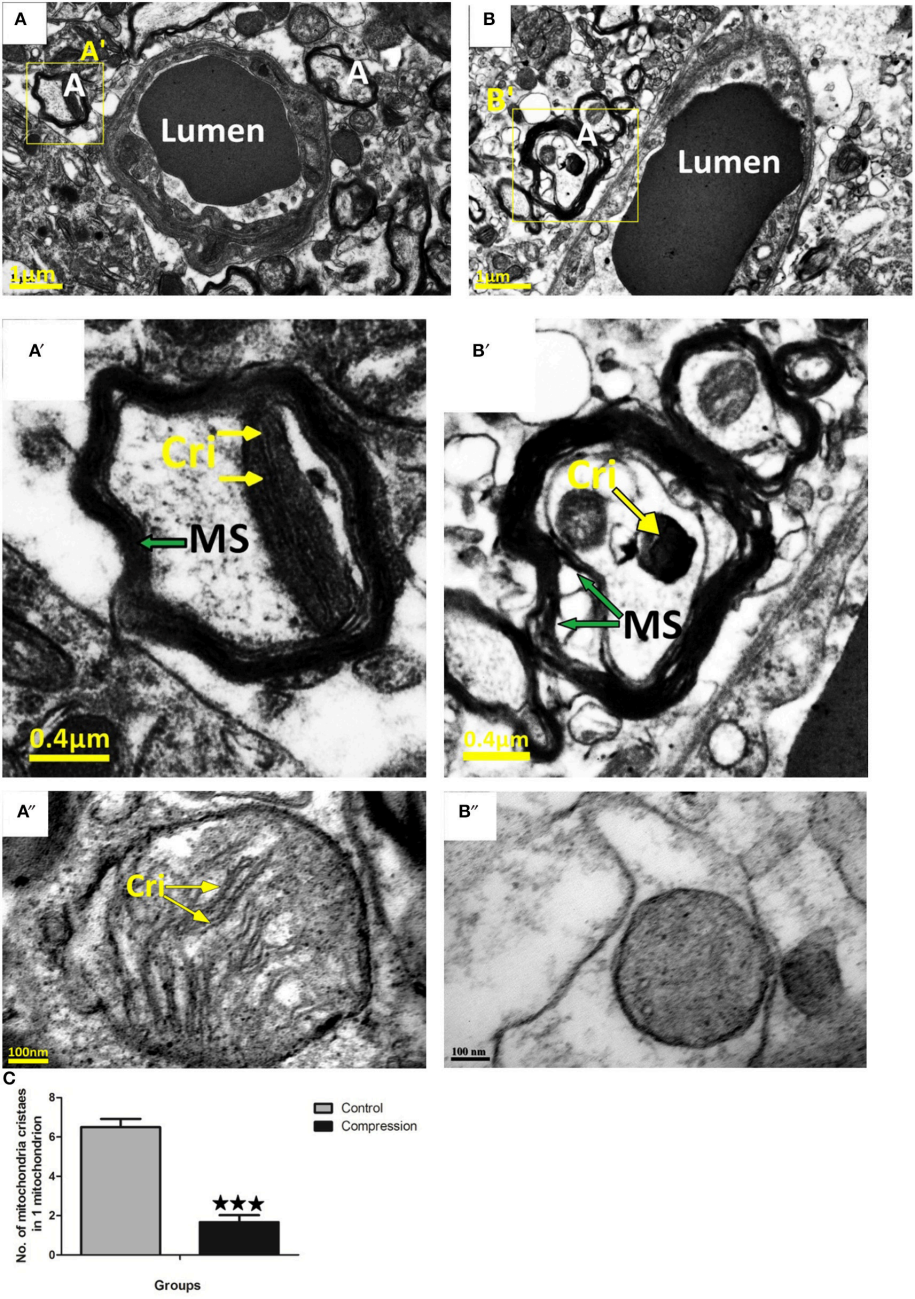
Myelin sheath of axon in control group was intact and homogeneous **(A,A**′**,A**″**)**, mitochondria in myelinated axons were normal. Loose myelin sheath and damaged mitochondria were observed in the compression group **(B,B**′**,B**″**)**. The numbers of mitochondria cristaes in 1 mitochondrion declined significantly after compression **(C)**, the number of mitochondria analyzed per group was 30, sampled from 12 rats per group. MS, Myelin Sheath; Cri, Mitochondria Cristae; A, Axons. ^***^P < 0.001 relative to controls.

In the control group, tightly packed myelinated axons filled the neuropil surrounding the capillaries (Figure 8A). At the same time, mitochondria with well-preserved cristae were seen in the neuronal cytoplasm. Compared with the control group, loose myelin sheaths and mitochondria with swollen cristae were seen in myelinated axons near capillaries (Figure 8B).

#### Neurons

**Figure 9:**
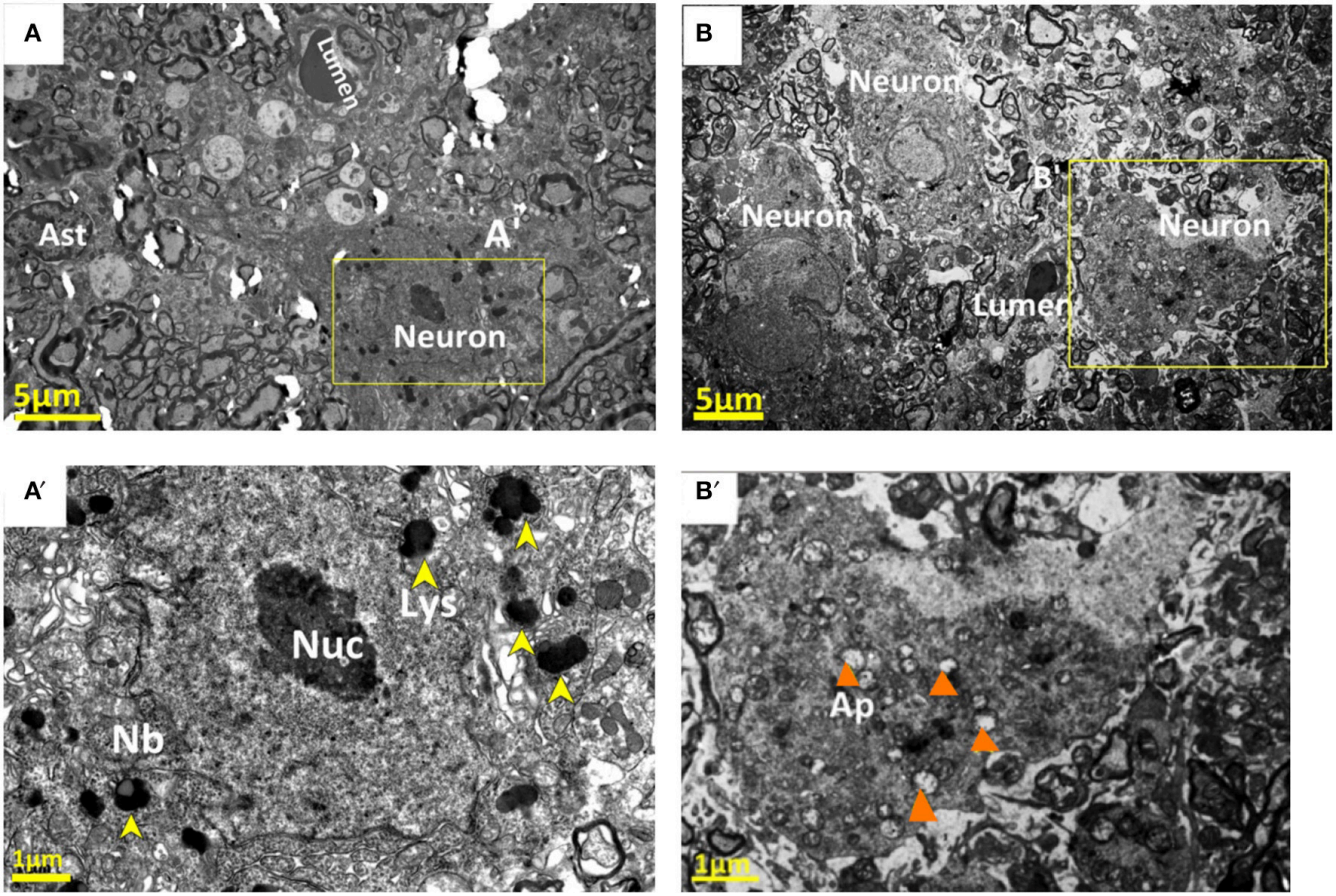
Normal nucleolus (Nuc), Nissl bodies (Nb), and lysosomes (Lys) were shown in the neuronal cytoplasm in the control group **(A,A**′**)**. Corresponding neurons in the compression group showed morphological signs of neurodegeneration, numerous phagolysosomes and autophagic vacuoles (Ap) were seen in the cytoplasm of neurons **(B,B**′**)**. The yellow arrowheads stand for “lysosomes (Lys)”; and the orange arrowheads for “autophagic vacuoles.”

Ultrastructural changes were also seen in the neurons contributing to the neurovascular unit. In the control group, Nissl bodies (Nb) and lysosomes (Lys) were equally distributed in the neuronal cytoplasm (Figures 9A,A′). After compression, these neurons showed morphological changes consistent with neurodegeneration, and often showed ultrastructural features of cellular death. Numerous phagolysosomes and autophagic vacuoles were seen in the cytoplasm of many neurons in the compressive group (Figure 9B′). In addition, certain neurons contained electron dense cytoplasm with nuclei separated by a lucid rim from the remainder of the cell. These cells are so-called pre-apoptotic neurons (Figure 9B). In addition to pre-apoptotic neurons and neurons undergoing autophagy, the analyzed specimens contained neurons with necrotic features, characterized by electron-lucent cytoplasm with a distinct nucleus containing loose, electron-lucent chromatin Figure 9B′.

#### Basement membrane (BM)

**Figure 10:**
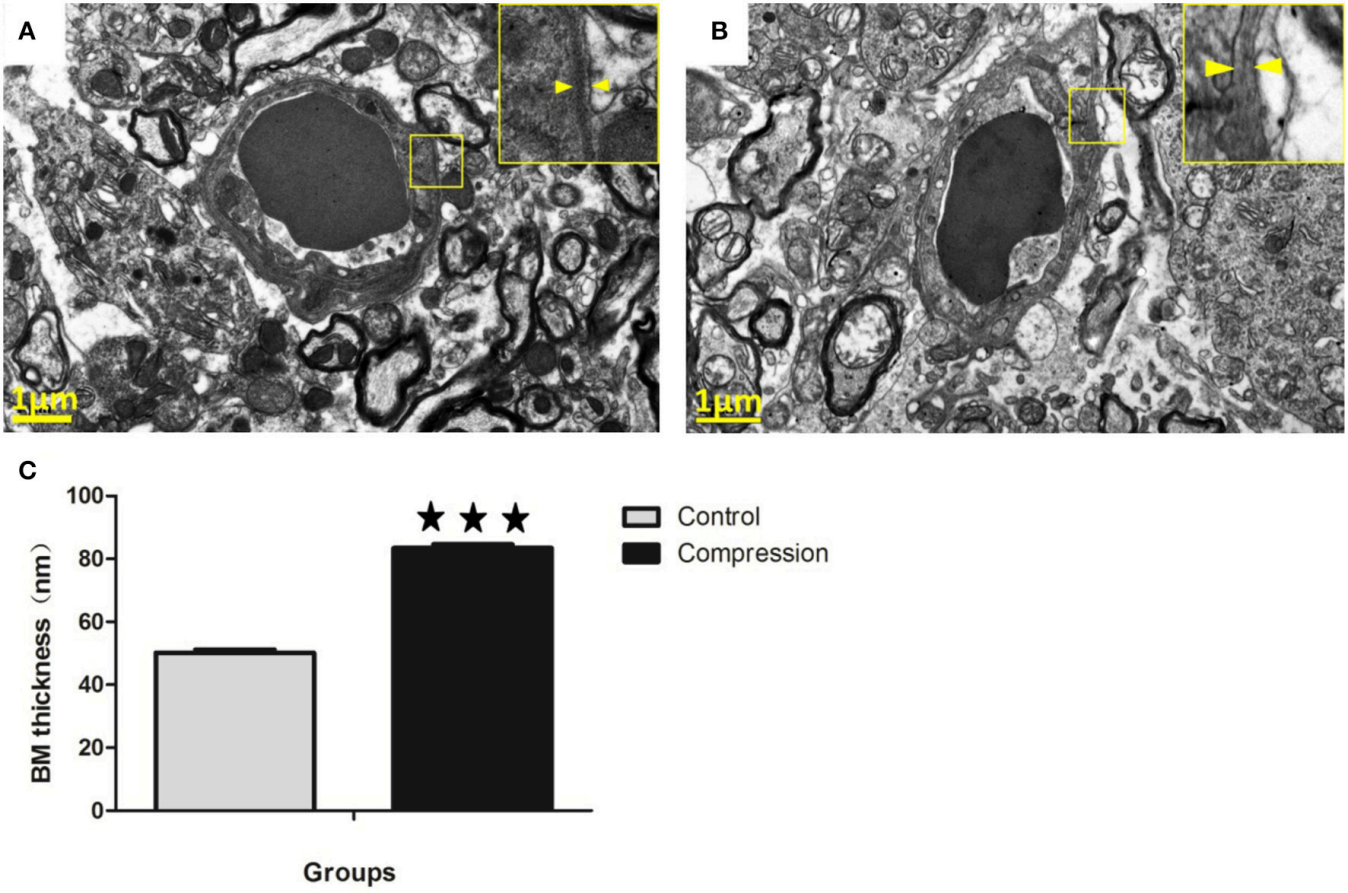
In the control group, microvessels were encapsulated with a thin BM **(A)**. In the compression group, the BM was thickened considerably, but with lower electron density **(B)**. Histograms show that BM thickness significantly increased in the compression group **(C)**. The number of capillaries analyzed per group was 30, sampled from 12 rats per group. ^***^P < 0.001 relative to controls.

In the control group, the capillaries consisted of a single layer of endothelial cells forming a lumen and a single layer of basement membrane surrounded by astrocytes (Figure 10A). However, the thickness of the basement membrane was greater in the compression group (Figures 10B,C). In some samples the capillaries consist of severely vacuolated endothelial cells surrounded by several layers of thickened basement membrane.

## Discussion

Focusing on neurovascular units is becoming a prerequisite for investigating the physiology and pathology of the central nervous system (CNS), contributing to discovery of therapeutic targets and drug candidates (Muoio et al., 2014). The normal physiological function of the spinal cord depends on the integrity of NVU and the regular interactions among various CNS cells. Neurons and astrocytes depend on the functional integrity of the vascular system to provide oxygen and energy (Deli et al., 2005). Meanwhile, the ultrastructure of the NVU has advantage in representing the integrity of spinal function and reflecting the intercellular communications of neurons with other type of cells under both physiological or pathological conditions, which may be more meaningful in the investigation of spinal cord diseases (such as CSM) and therapeutic drug screening. Our previous studies have proposed a chronic compressive cervical spinal cord injury rat model using a water-absorbent polymer that can provide controlled spinal cord compression (Long et al., 2013). The simplicity of operation, the high success rate, the high correlation with clinical characteristics, and the relative low cost make it an appropriate model for spinal cord injury studies. As we need to expose the phrenic nerve before tissue preparation, which may affect the forelimb behavior, we choose the BBB locomotor rating scale to test behavioral consequences of spinal cord injury (SCI) to the rat. Moreover, we found that some rats bit their own forelimb, which was supposed to the sign of paresthesia.

To our knowledge, NVU been analyzed in models of cerebral ischemia or ASL. Miyazaki et al. (2011) found that the damage in the NVU was more prominent in the outer side and preferentially in the anterior horn of ALS model mice. Nahirney PC (Nahirney et al., 2016) and his colleagues' results suggested that blood-brain barrier permeability in young and aged animals was mediated by transcellular pathways (caveolae/vacuoles), rather than tight junction loss. The blood-spinal cord barrier (BSCB), one NVU key players, may be affected during the development of SCI (Bartanusz et al., 2011).

Based on our survey of the literature, there is a paucity of data comparing changes in NVU ultrastructure after chronic cervical cord compression. Given this limitation, our discussion will focus primarily on showing the differences of our data with previous electron microscopy studies that subjected young adult animals to spinal cord injury or stroke. In general, ultrastructural studies have indicated that vessel permeability induced by ischaemia, hypoxia or other forms of injury is mediated primarily by a transcellular conduit system (Garbuzova-Davis et al., 2007; Kwon et al., 2009; Bauer et al., 2014; Figley et al., 2014). It should be noted that BSCB permeability could also be reliant on paracellular transport through TJ that normally binds endothelial cells together (Bartanusz et al., 2011). Ischaemia was first reported as one of the crucial pathophysiological mechanisms in CSM (Brain et al., 1948). Ischaemia-induced disruption of TJ has been reported by several studies which show a loss of TJ protein levels or a signal reduction in immunofluorescence assays of ischaemic tissues. Reconciling these competing views, a recent study by Knowland et al. (2014) suggested that the initial phases of BBB permeability following ischaemia and reperfusion in young animals are dependent on a caveolin-based transcytosis in the first 24 h which is accompanied by a disruption of TJs 2–3 days post-stroke. In our study, the ultrastructural analysis of young adult rats provided strong evidence that BSCB permeability on the 28th day after compression was associated with an increase in caveolae and vacuoles in the endothelium. While we are assuming the direction of vesicular movement (and hence permeability) based on static images, previous nanogold tracer experiments, as well as others (Shinnou et al., 1998; Chen et al., 2009; Krueger et al., 2013; Knowland et al., 2014; Reeson et al., 2015), have suggested that vesicles/vacuoles were likely moving blood-borne constituents from the luminal to abluminal side of the vascular endothelium (Ben-Zvi et al., 2014). Our data indicate that subtle TJ fluctuations can occur, as we did see small spaces at these interfaces which is also consistent with previous studies (Pluta et al., 1994; Bauer et al., 2014; Knowland et al., 2014), and may help explain previous reports about the loss of TJ protein. Pooling of fluid-filled spaces at the TJ seem to support the constant movement of vesicles/ vacuoles through the endothelium (Figure 4B′). However, the incidence of these partial disruptions was relatively low (20%) as most TJs were contiguous. It should be noted that complete uncoupling of the TJ from the luminal to abluminal side of the endothelium was never observed in the compressive vessels we sampled. Given the paucity of even partial TJ disruption and the near ubiquitous increase in vesicles and vacuoles in the endothelium, our data suggest that BSCB permeability (at the level of the endothelium) in the rat compressed spinal cord is likely based on transcellular mechanisms. The functional consequences of increased transcytotic activity are not entirely clear. One prediction is that increased transport of proteins and ions into endothelial cells, astrocytes, and pericytes may disrupt osmotic gradients and promote water influx and perivascular swelling.

Once the endothelial barrier has been broken, the basement membrane and pericytes can serve as another protective layer between blood and nervous tissue. Expansion of the basement membrane has been frequently described in cerebral ischaemia and aging (Nahirney et al., 2016). In our study, the expansion of the basement membrane region was strongly related to compression, since the basement membrane region in compromised vessels in the compressed core appeared much thicker than that found in the control group (Figure 10C). However, the electron density of BM was much lower. The cause or functional significance of this expansion is unclear, and the secretion of MMP-9 is highly correlated with the electron density of BM (Kwon et al., 2009).

Pericytes have been implicated as important players in the maintenance of BBB/BSCB integrity, given that pericyte loss in mutant mice leads to micro-aneurysms and increased vascular permeability (Armulik et al., 2010; Bell et al., 2010). Furthermore, pericytes can secrete enzymes, such as matrix metalloproteinase (Machida et al., 2015), that degrade the basement membrane and thus regulate BBB/BSCB permeability. One of the primary compression-induced effects in our study was swelling of pericytes and resulting increases in their coverage of the vascular endothelium was distinct from that in the control group. It is possible that the progressive increase in pericyte area and coverage after compression represents a compensatory mechanism to limit further BSCB permeability. Another interesting finding of our study was the increased presence of vesicles/vacuoles in pericytes. Transcellular movement of blood-borne protein through pericytes has been reported in non-ischaemic vessels (Broadwell et al., 1988). Our study extends this observation and suggests that chronic compression can upregulate constitutive activity of this transcellular pathway in pericytes.

Astrocyte endfeet are known to undergo significant swelling under ischaemic conditions (Shinnou et al., 1998; Ito et al., 2011). Our data corroborate this effect and confirm its persistence after compression. Mitochondria in astrocytes were also severely swollen and disrupted, and exhibited disorganized cristae (Ito et al., 2011). Interestingly, these enlarged astrocytes became highly enriched with glycogen granules after 28 days of compression. This observation is consistent with previous electron microscopic studies showing that glycogen granules are present after ischaemia (Kajihara et al., 2001). While the functional significance of these glycogen granules is unknown to date, they may reflect a reduction of enzymes that degrade glycogen, or increased synthesis of glycogen due to lower blood flow and elevated levels of glucose uptake/utilization in compression areas (Nedergaard et al., 1988).

We observed an increase of pre-apoptotic neurons in the gray matter beneath the compression site on the 28th day after compression. Pre-apoptotic neurons have been previously reported in response to mechanical trauma (Dietrich et al., 1994; Creeley et al., 2004; Lu et al., 2012). The pre-apoptotic neurons we observed typically had irregularly shaped nuclei, disorganized dendritic microtubule filaments, extensive phagolysosomes, and autophagic vacuoles, and swollen mitochondrial organelles. Some neurons also demonstrated increased levels of polyribosomes, indicating an increase in protein synthesis. Our data suggest that gray matter is susceptible to damage after 28 days of compression, which showed more prominent neuroglial cell changes at this time point after compression. The reason for this vulnerability may lie in the viscoelastic and cellular organization of these different cervical cord regions. Blood vessels are closely linked to neuronal activity. Thus, blood flow increases in response to local neuronal activation. However, the cellular mechanisms of this process are not completely understood. The breakdown of NVU is often followed by pathological changes in blood flow and perfusion pressure, but the mechanism of this process is not completely understood.

The number of degenerated axons was significantly increased in the compression group vs. the control group without any appreciable change in axonal density. These degenerative features were characterized by multifocal axonal injuries located around small blood vessels, and the loss of myelin and axons ranged from mild to moderate (Figure 8B). The mechanisms underlying axonal injury have been extensively reviewed (Buki and Povlishock, 2006; Smith et al., 2013; Kou and VandeVord, 2014). Axons are highly susceptible to mechanical stretching and shearing produced by rotational or linear rapid acceleration and deceleration forces during a direct impact at the point of compression. This susceptibility to injury is attributed to the viscoelastic properties of axon fibers and their high membrane-to-cytoplasm ratio, which makes them vulnerable to high rate of deformation damage. The total extent and magnitude of these axonal deformations after compression are unknown. We consider that the compromised axonal integrity in the cervical cord may therefore be one of the main causes for the impairment in neurobehavioral tasks seen in our rat model.

In summary, our imaging data reveal that NVU disruption in the compressive zone is associated with (1) a striking increase of putative transcytotic vesicles and vacuoles in the endothelium of the compression group, (2) sparse evidence for disruption of interendothelial TJs, (3) progressive swelling of pericytes with increased endothelial coverage, (4) expansion of the basement membrane region, (5) astrocyte swelling and mitochondrial disorganization, (6) progressive accumulation of glycogen granules in astrocytes at 28 d, and (7) degeneration of neuron and axon. These observations at the ultrastructural level should help guide future investigations attempting to ameliorate NVU dysfunction after chronic compression.

## Ethics statement

All described procedures were approved by the Research Ethics Committee of the First Affiliated Hospital of Sun yat-sen university and conducted in compliance with the Guide for the Care and Use of Laboratory Animals.

## Author contributions

HL and JX: Designed the experiments; JX, XC, and HY: Carried out experiments; JX and WC: Analyzed experimental results. YH and XW: Analyzed sequencing data and developed analysis tools; FL: Assisted with Illumina sequencing; JX, HL, and WC: Wrote the manuscript.

### Conflict of interest statement

The authors declare that the research was conducted in the absence of any commercial or financial relationships that could be construed as a potential conflict of interest.

## References

[B1] Armulik A. Genove G. Mae M. Nisancioglu M. H. Wallgard E. Niaudet C.. (2010). Pericytes regulate the blood-brain barrier. Nature 468, 557–561. 10.1038/nature09522 20944627

[B2] Bartanusz V. Jezova D. Alajajian B. Digicaylioglu M. (2011). The blood-spinal cord barrier: morphology and clinical implications. Ann. Neurol. 70, 194–206. 10.1002/ana.22421 21674586

[B3] Bauer H. C. Krizbai I. A. Bauer H. Traweger A. (2014). “You shall not pass”-tight junctions of the blood brain barrier. Front. Neurosci. 8:392. 10.3389/fnins.2014.00392 25520612 PMC4253952

[B4] Bell R. D. Winkler E. A. Sagare A. P. Singh I. LaRue B. Deane R.. (2010). Pericytes control key neurovascular functions and neuronal phenotype in the adult brain and during brain aging. Neuron 68, 409–427. 10.1016/j.neuron.2010.09.043 21040844 PMC3056408

[B5] Ben-Zvi A. Lacoste B. Kur E. Andreone B. J. Mayshar Y. Yan H.. (2014). Mfsd2a is critical for the formation and function of the blood-brain barrier. Nature 509, 507–511. 10.1038/nature13324 24828040 PMC4134871

[B6] Brain W. R. Knight G. C. Bull J. W. (1948). Discussion of rupture of the intervertebral disc in the cervical region. Proc. R. Soc. Med. 41, 509–516. 18877123 10.1177/003591574804100806 PMC2184583

[B7] Broadwell R. D. Balin B. J. Salcman M. (1988). Transcytotic pathway for blood-borne protein through the blood-brain barrier. Proc. Natl. Acad. Sci. U.S.A. 85, 632–636. 10.1073/pnas.85.2.632 2448779 PMC279605

[B8] Buki A. Povlishock J. T. (2006). All roads lead to disconnection?–Traumatic axonal injury revisited. Acta Neurochir. 148, 181–193; discussion: 193–194. 10.1007/s00701-005-0674-4 16362181

[B9] Chen B. Friedman B. Cheng Q. Tsai P. Schim E. Kleinfeld D.. (2009). Severe blood-brain barrier disruption and surrounding tissue injury. Stroke 40, e666–e674. 10.1161/STROKEAHA.109.551341 19893002 PMC2819286

[B10] Cheng X. Long H. Chen W. Xu J. Huang Y. Li F. (2015). Three-dimensional alteration of cervical anterior spinal artery and anterior radicular artery in rat model of chronic spinal cord compression by micro-CT. Neurosci. Lett. 606, 106–112. 10.1016/j.neulet.2015.08.050 26327142

[B11] Creeley C. E. Wozniak D. F. Bayly P. V. Olney J. W. Lewis L. M. (2004). Multiple episodes of mild traumatic brain injury result in impaired cognitive performance in mice. Acad. Emerg. Med. 11, 809–819. 10.1111/j.1553-2712.2004.tb00761.x 15289185

[B12] Deli M. A. Abraham C. S. Kataoka Y. Niwa M. (2005). Permeability studies on *in vitro* blood-brain barrier models: physiology, pathology, and pharmacology. Cell. Mol. Neurobiol. 25, 59–127. 10.1007/s10571-004-1377-8 15962509 PMC11529645

[B13] Dietrich W. D. Alonso O. Halley M. (1994). Early microvascular and neuronal consequences of traumatic brain injury: a light and electron microscopic study in rats. J. Neurotrauma 11, 289–301. 10.1089/neu.1994.11.289 7996583

[B14] Fehlings M. G. Wilson J. R. Karadimas S. K. Arnold P. M. Kopjar B. (2013). Clinical evaluation of a neuroprotective drug in patients with cervical spondylotic myelopathy undergoing surgical treatment: design and rationale for the CSM-Protect trial. Spine 38, S68–S75. 10.1097/BRS.0b013e3182a7e9b0 23962993

[B15] Figley S. A. Khosravi R. Legasto J. M. Tseng Y. F. Fehlings M. G. (2014). Characterization of vascular disruption and blood-spinal cord barrier permeability following traumatic spinal cord injury. J. Neurotrauma 31, 541–552. 10.1089/neu.2013.3034 24237182 PMC3949504

[B16] Frascarelli M. Oppido P. A. Rocchi L. Delfini R. D'Orazi G. (1990). Chronic damage after spinal trauma in rat: neurophysiological and ultrastructural investigations. J. Neurosurg. Sci. 34, 1–6. 2401908

[B17] Frontczak-Baniewicz M. Chrapusta S. J. Sulejczak D. (2011). Long-term consequences of surgical brain injury - characteristics of the neurovascular unit and formation and demise of the glial scar in a rat model. Folia Neuropathol. 49, 204–218. 22101954

[B18] Garbuzova-Davis S. Haller E. Saporta S. Kolomey I. Nicosia S. V. Sanberg P. R. (2007). Ultrastructure of blood-brain barrier and blood-spinal cord barrier in SOD1 mice modeling ALS. Brain Res. 1157, 126–137. 10.1016/j.brainres.2007.04.044 17512910

[B19] Hernandez-Chavarria F. (2002). [A view of tropical biology through the electron microscope]. Rev. Biol. Trop. 50, 927–940. 12947579

[B20] Herrera G. A. (1992). Microanalytical techniques and image analysis in the evaluation of immunogold-labeled specimens at the ultrastructural level. Ultrastruct. Pathol. 16, 127–135. 10.3109/01913129209074556 1557815

[B21] Ito U. Hakamata Y. Kawakami E. Oyanagi K. (2011). Temporary [corrected] cerebral ischemia results in swollen astrocytic end-feet that compress microvessels and lead to delayed [corrected] focal cortical infarction. J. Cereb. Blood Flow Metab. 31, 328–338. 10.1038/jcbfm.2010.97 20588315 PMC3049496

[B22] Kajihara H. Tsutsumi E. Kinoshita A. Nakano J. Takagi K. Takeo S. (2001). Activated astrocytes with glycogen accumulation in ischemic penumbra during the early stage of brain infarction: immunohistochemical and electron microscopic studies. Brain Res. 909, 92–101. 10.1016/S0006-8993(01)02640-3 11478925

[B23] Kalsi-Ryan S. Karadimas S. K. Fehlings M. G. (2013). Cervical spondylotic myelopathy: the clinical phenomenon and the current pathobiology of an increasingly prevalent and devastating disorder. Neuroscientist 19, 409–421. 10.1177/1073858412467377 23204243

[B24] Kaptanoglu E. Palaoglu S. Surucu H. S. Hayran M. Beskonakli E. (2002). Ultrastructural scoring of graded acute spinal cord injury in the rat. J. Neurosurg. 97, 49–56. 10.3171/spi.2002.97.1.0049 12120651

[B25] Karadimas S. K. Erwin W. M. Ely C. G. Dettori J. R. Fehlings M. G. (2013a). Pathophysiology and natural history of cervical spondylotic myelopathy. Spine 38, S21–S36. 10.1097/BRS.0b013e3182a7f2c3 23963004

[B26] Karadimas S. K. Gatzounis G. Fehlings M. G. (2015). Pathobiology of cervical spondylotic myelopathy. Eur. Spine J. 24(Suppl. 2), 132–138. 10.1007/s00586-014-3264-4 24626958

[B27] Karadimas S. K. Moon E. S. Yu W. R. Satkunendrarajah K. Kallitsis J. K. Gatzounis G.. (2013b). A novel experimental model of cervical spondylotic myelopathy (CSM) to facilitate translational research. Neurobiol. Dis. 54, 43–58. 10.1016/j.nbd.2013.02.013 23466695

[B28] Knowland D. Arac A. Sekiguchi K. J. Hsu M. Lutz S. E. Perrino J.. (2014). Stepwise recruitment of transcellular and paracellular pathways underlies blood-brain barrier breakdown in stroke. Neuron 82, 603–617. 10.1016/j.neuron.2014.03.003 24746419 PMC4016169

[B29] Kou Z. VandeVord P. J. (2014). Traumatic white matter injury and glial activation: from basic science to clinics. Glia 62, 1831–1855. 10.1002/glia.22690 24807544

[B30] Krueger M. Hartig W. Reichenbach A. Bechmann I. Michalski D. (2013). Blood-brain barrier breakdown after embolic stroke in rats occurs without ultrastructural evidence for disrupting tight junctions. PLoS ONE 8:e56419. 10.1371/journal.pone.0056419 23468865 PMC3582567

[B31] Kubota Y. (2015). New developments in electron microscopy for serial image acquisition of neuronal profiles. Microscopy 64, 27–36. 10.1093/jmicro/dfu111 25564566

[B32] Kwon I. Kim E. H. del Zoppo G. J. Heo J. H. (2009). Ultrastructural and temporal changes of the microvascular basement membrane and astrocyte interface following focal cerebral ischemia. J. Neurosci. Res. 87, 668–676. 10.1002/jnr.21877 18831008 PMC2711693

[B33] Long H. Q. Li G. S. Lin E. J. Xie W. H. Chen W. L. Luk K. D.. (2013). Is the speed of chronic compression an important factor for chronic spinal cord injury rat model? Neurosci. Lett. 545, 75–80. 10.1016/j.neulet.2013.04.024 23632138

[B34] Long H. Q. Xie W. H. Chen W. L. Xie W. L. Xu J. H. Hu Y. (2014). Value of micro-CT for monitoring spinal microvascular changes after chronic spinal cord compression. Int. J. Mol. Sci. 15, 12061–12073. 10.3390/ijms150712061 25003643 PMC4139829

[B35] Lu J. Ng K. C. Ling G. Wu J. Poon D. J. Kan E. M.. (2012). Effect of blast exposure on the brain structure and cognition in *Macaca fascicularis*. J. Neurotrauma 29, 1434–1454. 10.1089/neu.2010.1591 21639720

[B36] Machida T. Takata F. Matsumoto J. Takenoshita H. Kimura I. Yamauchi A.. (2015). Brain pericytes are the most thrombin-sensitive matrix metalloproteinase-9-releasing cell type constituting the blood-brain barrier *in vitro*. Neurosci. Lett. 599, 109–114. 10.1016/j.neulet.2015.05.028 26002077

[B37] Miyazaki K. Ohta Y. Nagai M. Morimoto N. Kurata T. Takehisa Y.. (2011). Disruption of neurovascular unit prior to motor neuron degeneration in amyotrophic lateral sclerosis. J. Neurosci. Res. 89, 718–728. 10.1002/jnr.22594 21337372

[B38] Muoio V. Persson P. B. Sendeski M. M. (2014). The neurovascular unit - concept review. Acta Physiol. 210, 790–798. 10.1111/apha.12250 24629161

[B39] Nahirney P. C. Reeson P. Brown C. E. (2016). Ultrastructural analysis of blood-brain barrier breakdown in the peri-infarct zone in young adult and aged mice. J. Cereb. Blood Flow Metab. 36, 413–425. 10.1177/0271678X15608396 26661190 PMC4759675

[B40] Nedergaard M. Jakobsen J. Diemer N. H. (1988). Autoradiographic determination of cerebral glucose content, blood flow, and glucose utilization in focal ischemia of the rat brain: influence of the plasma glucose concentration. J. Cereb. Blood Flow Metab. 8, 100–108. 10.1038/jcbfm.1988.13 3339100

[B41] Pluta R. Lossinsky A. S. Wisniewski H. M. Mossakowski M. J. (1994). Early blood-brain barrier changes in the rat following transient complete cerebral ischemia induced by cardiac arrest. Brain Res. 633, 41–52. 10.1016/0006-8993(94)91520-2 8137172

[B42] Ramadan W. S. Abdel-Hamid G. A. Al-Karim S. Abbas A. T. (2017). Histological, immunohistochemical and ultrastructural study of secondary compressed spinal cord injury in a rat model. Folia Histochem. Cytobiol. 55, 11–20. 10.5603/FHC.a2017.0001 28509312

[B43] Reeson P. Tennant K. A. Gerrow K. Wang J. Weiser Novak S. Thompson K.. (2015). Delayed inhibition of VEGF signaling after stroke attenuates blood-brain barrier breakdown and improves functional recovery in a comorbidity-dependent manner. J. Neurosci. 35, 5128–5143. 10.1523/JNEUROSCI.2810-14.2015 25834040 PMC6705411

[B44] Shinnou M. Ueno M. Sakamoto H. Ide M. (1998). Blood-brain barrier damage in reperfusion following ischemia in the hippocampus of the Mongolian gerbil brain. Acta Neurol. Scand. 98, 406–411. 10.1111/j.1600-0404.1998.tb07322.x 9875619

[B45] Smith D. H. Johnson V. E. Stewart W. (2013). Chronic neuropathologies of single and repetitive TBI: substrates of dementia? Nat. Rev. Neurol. 9, 211–221. 10.1038/nrneurol.2013.29 23458973 PMC4513655

[B46] Smith P. M. Jeffery N. D. (2006). Histological and ultrastructural analysis of white matter damage after naturally-occurring spinal cord injury. Brain Pathol. 16, 99–109. 10.1111/j.1750-3639.2006.00001.x 16768749 PMC8095982

[B47] Song H. Fang X. Wen M. Yu F. Gao K. Sun C.. (2015). Role of MK2 signaling pathway in the chronic compression of cervical spinal cord. Am. J. Transl. Res. 7, 2355–2363. 26807183 PMC4697715

